# Positive emotional responses to anthropomorphic virtual learning assistants among university students with different levels of loneliness

**DOI:** 10.3389/fpsyg.2026.1758999

**Published:** 2026-09-07

**Authors:** Yuyang Tian, Ye Qiu

**Affiliations:** School of Arts and Media, Nanning College of Technology, Nanning, Guangxi, China

**Keywords:** anthropomorphic design, loneliness, positive emotions, social presence, virtual learning assistants

## Abstract

Loneliness is increasingly prevalent among university students, posing substantial challenges to their mental health and academic performance. From a positive psychology perspective, cultivating positive emotional experiences may strengthen psychological resilience and wellbeing, particularly among individuals experiencing loneliness. Although virtual learning assistants (VLAs) are widely used in higher education, prior research has focused primarily on their functionality and learning outcomes, leaving their emotional effects, underlying mechanisms, and boundary conditions insufficiently understood. Drawing upon Social Response Theory, Emotional Contagion Theory, and Social Surrogacy Theory, this study examines how anthropomorphic VLAs influence university students' positive emotions through social presence and whether loneliness moderates this process. Across four controlled experiments, anthropomorphic design incorporating visual and verbal cues was compared with non-anthropomorphic design in terms of social presence and positive emotions. The results showed that anthropomorphic VLAs elicited greater social presence and stronger positive emotions than non-anthropomorphic VLAs. Social presence mediated the effect of anthropomorphic design on positive emotions, while loneliness strengthened its effects on both social presence and positive emotions. These findings extend VLA research from an instrumental to an affective perspective, clarify the social-psychological mechanism and boundary condition underlying anthropomorphic design effects, and inform the design of emotionally supportive educational technologies for students with higher levels of loneliness.

## 1 Introduction

University represents a major life transition involving substantial changes in students' lifestyles and social relationships (Tian et al., 2025a). When adequate social support is lacking, these changes may heighten feelings of loneliness (Ellard et al., 2023). Evidence from different countries indicates that loneliness is widespread among university students. For example, 32.4% of students in Germany reported moderate loneliness and 3.2% reported severe loneliness (Diehl et al., 2018). while studies conducted in the United Kingdom, Jordan, and Indonesia have similarly documented high levels of loneliness among young people and university students (Ellard et al., 2023; Indrayanti et al., 2025). A large cross-national study spanning 62 countries further underscores the global relevance of loneliness among university students (Aristovnik et al., 2020).

Loneliness refers to the perceived discrepancy between an individual's desired and actual social relationships (Hickin et al., 2021). and is commonly characterized by feelings of isolation, alienation, and lack of companionship (Park et al., 2020). Among university students, loneliness is associated with anxiety, stress, depression, maladaptive coping, sleep disturbances, and reduced life satisfaction (Diehl et al., 2018; Chen et al., 2013). It also impairs cognitive functioning and and social interaction, thereby reducing academic engagement (Le et al., 2019; Mizani et al., 2022).

From a Positive psychology perspective, positive emotional experiences may strengthen psychological resilience and wellbeing, particularly among individuals experiencing loneliness (Fredrickson, 2000). However, limited research has examined whether technologies embedded in students' everyday learning environments can foster positive emotional experiences. Virtual learning assistants (VLAs), which are increasingly integrated into university students' daily learning activities, may provide a promising context for addressing this gap.

These assistants use rapid-response and natural language processing capabilities to provide personalized learning support, facilitate communication, and enhance learning engagement (Gubareva and Lopes, 2020). They take various forms, including mobile applications, intelligent learning platforms, and embedded virtual agents (Raisa et al., 2025; Todericiu, 2025). Despite growing scholarly interest, prior research has focused primarily on their functional capabilities and learning-related outcomes, with limited attention to how their design features shape students' emotional experiences, particularly among those experiencing loneliness.

Anthropomorphism, is a design strategy that endows products, brands, chatbots, and virtual agents with human-like characteristics, such as emotions, personality, and behaviors thereby encouraging users to perceive them as social entities (Alabed et al., 2022). These characteristics are typically conveyed through visual cues such as facial expressions, gestures) and verbal cues such as humorous or warm communication styles; Chen et al., 2024). Previous research suggests that anthropomorphic design elicits stronger positive emotional responses in users than non-anthropomorphic ones (Ma et al., 2023). Notably, individuals with higher levels of loneliness tend to show responsive to anthropomorphic agents because unmet social needs motivate them to seek companionship from nonhuman entities (Dang and Liu, 2023; Chen et al., 2017). However, the emotional effects of anthropomorphic design remain underexplored in the VLA context.

Furthermore, social presence, as a key psychological perception in mediated interactions, plays a key role in eliciting positive emotions in anthropomorphic contexts (Konya-Baumbach et al., 2023). However, its mediating role in the relationship between anthropomorphic VLA design and positive emotions remains underexplored. Moreover, whether loneliness strengthens the effects of anthropomorphic VLA design on social presence and positive emotions, and whether this pattern generalizes across visual and verbal cues, remain unclear.

Building on this background, this study develops an integrated model in which anthropomorphic VLA design influences university students' positive emotions through social presence, while loneliness moderates its effects on social presence and positive emotions. Across four controlled experiments, the proposed relationships are examined using anthropomorphic visual and verbal cues. This study makes three contributions. First, it extends VLA research beyond functional capabilities and learning-related outcomes by examining students' positive emotional experiences. Second, it identifies social presence as a key psychological mechanism underlying the emotional effects of anthropomorphic VLA design. Third, it clarifies loneliness as a boundary condition and examines the robustness of the proposed process across different anthropomorphic cues. Together, these contributions integrate anthropomorphic design, social presence, positive emotions, and loneliness within a unified framework for virtual learning environments.

## 2 Theoretical background and research hypotheses

### 2.1 The effect of anthropomorphic design on university students' positive emotions

Anthropomorphism is defined as “the tendency to imbue the real or imagined behavior of nonhuman agents with human-like characteristics, motivations, intentions, or emotions” (Epley et al., 2007). The three-factor theory of anthropomorphism further explains the psychological mechanisms that elicit this tendency (Ding et al., 2021). First, elicited agent knowledge refers to individuals' use of human related knowledge and cognitive frameworks to interpret unfamiliar non-human agents. When a virtual learning assistant (VLA) displays human-like facial expressions, emotions, or behavioral cues, users are more likely to attribute human-like traits and mental states to it, thereby eliciting emotional responses. Second, effectance motivation reflects individuals' desire to understand, predict, and interact effectively with their environment. By making a VLA appear more familiar, understandable, and predictable, anthropomorphic design may reduce uncertainty during the learning process and foster more positive emotional experiences. Finally, social motivation refers to individuals' intrinsic need for social connection. This motivation may encourage users to perceive anthropomorphic VLAs as socially responsive interaction partners, thereby strengthening perceived connection and facilitating positive emotional responses.

From the perspective of utilization theory users rely on observable design features to infer the social and emotional attributes of technological agents, with anthropomorphic features commonly conveyed through visual and verbal cues (Tian et al., 2025b; Zhu et al., 2020). Visual cues such as human-like facial expressions, avatars, gestures, and movements can make artificial agents appear more socially engaging and emotionally expressive, thereby strengthening users' emotional attachment and favorable evaluations (Khan et al., 2023; Mishra and Mehta, 2023). Meta-analytic evidence further indicates that visual anthropomorphic cues enhance emotional engagement in human–computer interaction (Zhang et al., 2024) with similar effects observed in virtual-agent research (Munnukka et al., 2022).

Anthropomorphic verbal cues characterized by warmth and humor can enhance users' perceptions of anthropomorphism and activate social responses similar to those elicited in human interactions (Janson, 2023). Such interactions may foster trust and psychological closeness, thereby promoting positive emotional responses (Yan et al., 2024). Consistent with social response theory, language serves as a direct social cue through which artificial agents can elicit emotional resonance and interpersonal perceptions Wu et al., (2023). (Brännström et al. 2024) further introduced the concept of the “perception of computational empathy,” suggesting that users' perceptions of understanding, care, and empathic responsiveness can emerge from a chatbot's linguistic and interaction design. Thus, warm and humorous anthropomorphic verbal cues may lead users to perceive a virtual learning assistant as understanding and caring, thereby strengthening social connection and eliciting positive emotional responses.

Moreover, emotional contagion theory, suggests that emotions can be transmitted through social interaction (Herrando and Constantinides, 2021). Recent research indicates that similar affective responses may also emerge in human–AI interactions, as users can respond to the emotional cues expressed by artificial agents

Li et al. (2024a) For example, positive emotions conveyed by anthropomorphic chatbots may elicit corresponding emotional responses among users (Han et al., 2023). through human-like visual or verbal cues, anthropomorphic design can enhances the perceived warmth and familiarity of artificial intelligence (Liu et al., 2024). Therefore, warmth and humor expressions used by virtual learning assistant may reduce psychologically distance strengthen perceived affinity and foster positive emotional responses.

Based on the above reasoning, this study proposes that anthropomorphic Virtual Learning Assistants (VLAs) through both visual and verbal cues, may enhance positive emotional experiences, university students' emotional responses. Thus, we propose Hypothesis H1:

H1: Anthropomorphic design in virtual learning assistants has a significant positive effect on university students' positive emotions.
H1a: Anthropomorphic design (visual cues) in virtual learning assistants has a significant positive effect on university students' positive emotions.
H1b: Anthropomorphic design (verbal cues) in virtual learning assistants has a significant positive effect on university students' positive emotions.

### 2.2 The mediating effect of social presence

Social presence is defined as the “sense of being with another” (Biocca et al., 2003), referring to the extent to which individuals experience another social entity including non-human or artificial agent as present within a mediated environment (Van Der Stap et al., 2024). As a critical factor predicting users' psychological and behavioral responses, social presence plays a particularly vital role in the context of anthropomorphic design (Kim et al., 2020). It reflects the degree of warmth, intimacy, and authenticity evoked by anthropomorphic media or design cues, thereby enhancing users' emotional engagement and willingness to interact (Adam et al., 2021).

Anthropomorphism refers to the tendency to attribute human characteristics, emotions, or intentions to non-human entities whereas anthropomorphic design is considered an design strategies for enhancing users' parasocial interaction (Haugeland et al., 2022). Previous studies have shown that when technological objects display human-like traits, consumers tend to anthropomorphize them unconsciously, triggering social responses (Jeong et al., 2022). For instance, anthropomorphic cues in chatbot design, such as human-like names, avatars, emojis, and forms of address can effectively strengthen users' perceptions of social responsiveness and interpersonal engagement (Yu and Zhao, 2024). This effect can be explained by the Computers Are Social Actors (CASA) theory, which suggests that people automatically apply social rules and expectations derived from human interactions when engagement with virtual agents (Park et al., 2023), Consequently, users may experience the interaction as more personal and socially present (Xie et al., 2025).

Therefore, this study proposes that users perceive social presence depends partly on the extent to which a virtual learning assistant to convey anthropomorphic cues. when a virtual learning assistant exhibits human-like features such as facial expressions, tone of voice, gestures, friendliness, professionalism, and appearance, it can effectively simulate social interactions and enhance users' perception of social presence. Accordingly, Hypothesis H2 is proposed.

H2: Anthropomorphic design in virtual learning assistants has a significant positive effect on social presence.
H2a: Anthropomorphic design (visual cues) in virtual learning assistants has a significant positive effect on social presence.
H2b: Anthropomorphic design (verbal cues) in virtual learning assistants has a significant positive effect on socialpresence.

according to Social Response Theory, people tend to respond to anthropomorphic technologies in ways similar to how they respond during interpersonal interactions (Nass and Moon, 2000). When a virtual learning assistant displays human-like features, users may apply social expectations and interaction norms typically associated with other people, thereby perceiving the system as more socially responsive and interpersonally engaging (Xu et al., 2022). Such perceptions can strengthen social presence and, in turn, foster positive emotional responses.

Previous research suggests that when users perceive a technological agent as possessing human-like characteristics, they are more likely to regard it as a socially interactive entity, and develop stronger emotional attachments (Williamson and Szocs, 2021). This perception may increase users' sense of familiarity and reduce uncertainty during interaction, emotional responses thereby shaping more favorable emotional (Heerink et al., 2010). social presence elicited by anthropomorphic design has been associated with greater trust in artificial agents (Jiang et al., 2023). Particularly In online or virtual environments a stronger sense of social presence may enhance users' positive emotional responses to anthropomorphic agents (Lim et al., 2021).

Therefore, this study proposes that when a Virtual Learning Assistant (VLA) incorporates anthropomorphic design, users may perceive it not merely as a functional tool but as a socially responsive interaction partner. This perception can strengthen social presence and, in turn, enhances university students' positive emotions. Based on this reasoning, Hypothesis H3 is proposed.

H3: Social presence mediates the relationship between anthropomorphic design in virtual learning assistants and university students' positive emotions.
H3a: Social presence mediates the relationship between anthropomorphic design (visual cues) in virtual learning assistants and university students' positive emotions.
H3b: Social presence mediates the relationship between anthropomorphic design (verbal cues) in virtual learning assistants and university students' positiveemotions.

### 2.3 The moderating effect of loneliness

Loneliness is a subjective emotional state, commonly defined as an individual's perception of insufficient social connection or unmet social needs (Barjaková et al., 2023). individuals experiencing higher levels of loneliness typically have stronger needs and may therefore be more receptive to technologies that convey companionship and social responsiveness (Herbener and Damholdt, 2025). From the perspective of the Three-Factor Theory of Anthropomorphism, heightened sociality motivation increases individuals' tendency to seek human-like qualities in nonhuman agents (Yang et al., 2020). When needs for belonging and social connection remain unmet, lonely individuals may become more attentive and responsive to anthropomorphic cues (Zhang et al., 2023). Such cues can provide symbolic forms of social connections and temporarily support users' feelings of interpersonal engagemen (Yang et al., 2025b). Thus, loneliness may increase users' sensitivity to the anthropomorphic visual and verbal cues embedded in virtual learning assistants, thereby strengthening their perceptions of social presence and their positive emotional responses.

Social Surrogacy Theory suggests that when opportunities for meaningful social interaction are limited or existing relationships fail to adequately satisfy individuals' needs, people turn to media figures, fictional characters, or other symbolic entities as temporary sources of social connection (Mayor Poupis et al., 2021). Such surrogates may include preferred television programs, literature, music, public figures, or even particular foods and consumer goods (Feng, 2016). In this context, anthropomorphic technologies may provide cues of social responsiveness and interpersonal engagement that are particularly salient to individuals experiencing loneliness (Berridge et al., 2023).

In addition, according to Media Richness Theory, anthropomorphic verbal cues increase communication richness by conveying socially and emotionally relevant information, thereby strengthening users' perception of social presence (Miao et al., 2022). This heightened social presence is particularly salient to individuals experiencing loneliness, who have stronger unmet needs for social connection (Li et al., 2024b). Empirical research indicates that warm and humorous anthropomorphic dialogue can convey personality and emotional qualities while strengthening users' sense of social connection with technological agents (Li and Wang, 2023). This effect is partly attributable to the ability of language to evoke psychological closeness and perceived warmth (An et al., 2025). Moreover, natural and informal communication styles can strengthen users' perception of social presence of by making interactions feel more conversational and interpersonally engaging (Kim et al., 2021), thereby creating aa sense of dialogue between consumers and technological agents (Lin, 2025).

Therefore, this study proposes that students experiencing higher levels of loneliness, driven by a stronger desire for social belonging and emotional connection, are more sensitive to the visual and verbal cues conveyed by anthropomorphic design. Consequently, anthropomorphic virtual learning assistants are expected to elicit stronger perceptions of social presence and more positive emotional responses among these students. Accordingly, Hypotheses H4 and H5 are proposed.

H4: Loneliness moderates the relationship between anthropomorphic design in virtual learning assistants and social presence.
H4a: Loneliness moderates the relationship between anthropomorphic design (visual cues) in virtual learning assistants and social presence.
H4b: Loneliness moderates the relationship between anthropomorphic design (verbal cues) in virtual learning assistants and social presence.
H5: Loneliness moderates the relationship between anthropomorphic design in virtual learning assistants and university students' positive emotions.
H5a: Loneliness moderates the relationship between anthropomorphic design (visual cues) in virtual learning assistants and university students' positive emotions.
H5b: Loneliness moderates the relationship between anthropomorphic design (verbal cues) in virtual learning assistants and university students' positiveemotions.

## 3 Overview of studies

**Figure 1:**
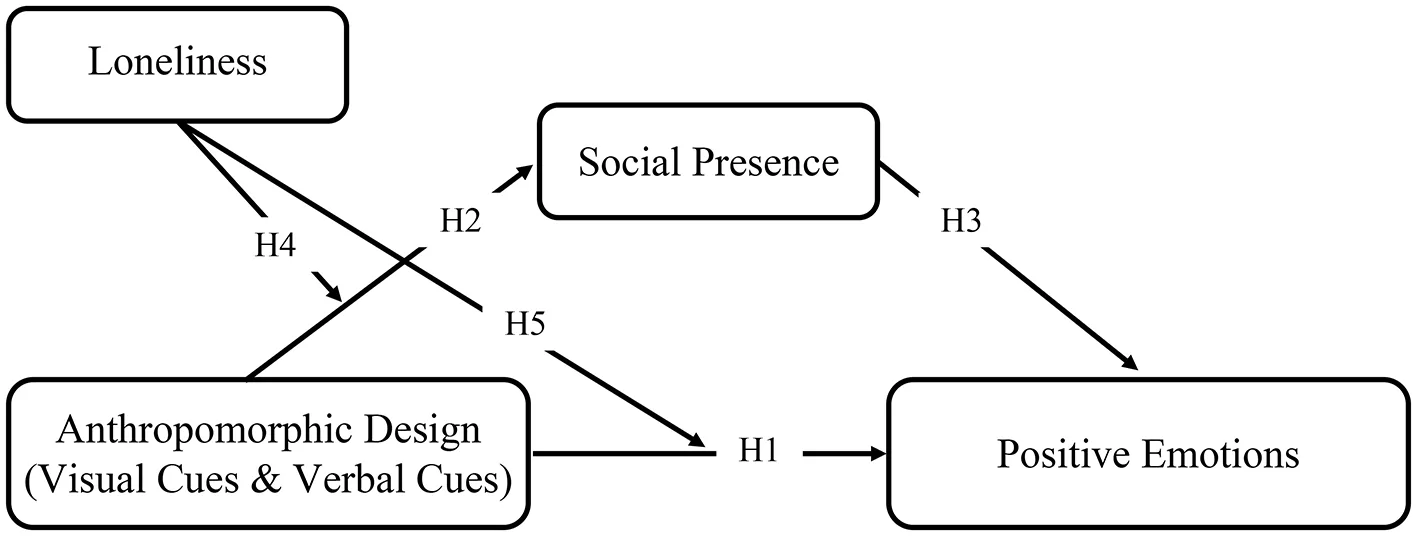
Illustrates the conceptual framework of this study, which was empirically tested through four experimental studies to verify all proposed hypotheses.

Specifically, Study 1a examines the effect of anthropomorphic design (visual cues) in virtual learning assistants on university students' positive emotions and explores the mediating role of social presence, thereby testing Hypotheses H1a, H2a, and H3a. Study 1b further investigates anthropomorphic design verbal cues), verifying its effect on students' positive emotions and the mediating effect of social presence, thus testing Hypotheses H1b, H2b, and H3b. Building on the first two experiments, Study 2a introduces loneliness as a moderator to examine whether it moderates the effects of anthropomorphic design (visual cues) on social presence and positive emotions, thereby testing Hypotheses H4a and H5a. Finally, Study 2b examines whether loneliness moderates the effects of anthropomorphic design (verbal cues) on social presence and positive emotions, thereby testing Hypotheses H4b and H5b (see Figure 1).

## 4 Study

The anthropomorphic manipulation used across the four studies were developed with reference to Aggarwal and McGill (2007) and refined based on subsequent research.

Two types of anthropomorphic cues were examined: visual cues involving human-like appearance characteristics and verbal cues involving human-like linguistic expressions. These manipulations enabled us to examine whether the effects of anthropomorphic virtual learning assistants on students' positive emotions, through perceived social presence, were consistent across different forms of anthropomorphic design. The later studies further examined loneliness as a continuous moderator of these relationships.

### 4.1 Study1a

Study 1a employed a one-factor between-subjects design (anthropomorphic visual cues: anthropomorphic vs. non-anthropomorphic). We predicted that university students in the anthropomorphic visual-cue condition would experience significantly higher levels of positive emotions than those in the non-anthropomorphic condition, and that social presence would mediate this relationship.

#### 4.1.1 Method and procedure

This study adopted a quantitative experimental design involving 118 university students (53% female; *M* = 22.05, SD = 3.55) from Shenzhen, Guangdong, China. Data were collection between January 29, 2025 and February 15, 2025. All participants received monetary compensation for their participation. and were randomly assigned to either the anthropomorphic design (visual cues) or non-anthropomorphic condition.

#### 4.1.2 Stimuli and manipulation

Study 1a used two versions of a virtual learning assistant icon embedded in the interface of a mock language-translation application. In the anthropomorphic condition, the icon incorporated human-like visual features, including human-like facial features (eyes, eyebrows, a smiling mouth), a thumbs-up gesture, and a headset with a microphone, creating a human-like visual appearance. In the non-anthropomorphic condition, the icon retained the same basic shape and functional symbolism but contained no human-like facial features and displayed a neutral, non-human appearance. The two versions were identical in dimensions, color palette, background, position, application label, and image resolution. After viewing the stimuli, participants completed the questionnaire. All scales were translated from English into Chinese and independently back-translated by bilingual researchers to ensure conceptual equivalence.

Participants assessed the perceived anthropomorphism of the virtual learning assistant using a four-item scale adapted from Aggarwal and McGill (2007), which has been widely applied in anthropomorphic artificial intelligence research (Affandi et al., 2025). The items were: “The virtual learning assistant looks like a person,” “The virtual learning assistant has a human-like expression,” “The virtual learning assistant has a human-like appearance,” and “The virtual learning assistant has come alive.” (Cronbach's alpha = 0.871).

Subsequently, positive emotions were assessed using six items adapted from the scale developed by Hsieh et al. (2021). The items were “The virtual learning assistant made me feel pleasure,” “happy,” “satisfied,” “active,” “excited,” and “hopeful” (Cronbach's alpha = 0.912). Finally, participants completed a five-item social presence scale adapted from Gao et al. (2017). The items were “There is a sense of human contact with the virtual learning assistant,” “There is a sense of personalness in the virtual learning assistant,” “There is a sense of sociability with the virtual learning assistant,” “There is a sense of human warmth in the virtual learning assistant,” and “There is a sense of human sensitivity in the virtual learning assistant” (Cronbach's alpha = 0.841). The measures demonstrated satisfactory reliability, convergent validity, and discriminant validity. Detailed CR, AVE, and HTMT results are reported in the Supplementary material.

#### 4.1.3 Result

Manipulation Check. An independent sample *t-*test was conducted to examine the effectiveness of the anthropomorphic design (visual cues) manipulation, with design type (anthropomorphic vs. non-anthropomorphic) as the independent variable and perceived anthropomorphism as the dependent variable. Participants in the anthropomorphic condition rated the virtual learning assistant as significantly more human-like (M = 3.93, SD = 1.086) than those in the non-anthropomorphic condition (*M* = 2.729, *SD* = 0.769), t (116) = −6.938, *p* < 0.001, *d* = 1.342, confirming the success effectiveness of the anthropomorphic manipulation.

#### 4.1.4 Positive emotions

To examine the effect of anthropomorphic design (visual cues) on university students' positive emotions, an independent sample *t*-test was conducted. Results showed that participants in the anthropomorphic condition reported significantly higher levels of positive emotions (M = 4.3 16, SD = 0.381) than those in the non-anthropomorphic condition (M = 2.3 75, SD = 0.901), t (116) = −14.053, *p* < 0.001, d = 2. 59. Thus, H1a was supported.

#### 4.1.5 Social presence

The analysis revealed that participants in the anthropomorphic design condition reported significantly higher levels of social presence (M = 4. 19, SD = 0.489) than those in the non-anthropomorphic condition (M = 2.969, SD = 1.128), t (116) = −7.086, *p* < 0.001, d = 1.411. This statistically significant difference indicates that the anthropomorphic virtual learning assistant with visual cues effectively enhanced students' sense of social presence, thereby supporting H2a.

#### 4.1.6 Mediation analysis

**Figure 2:**
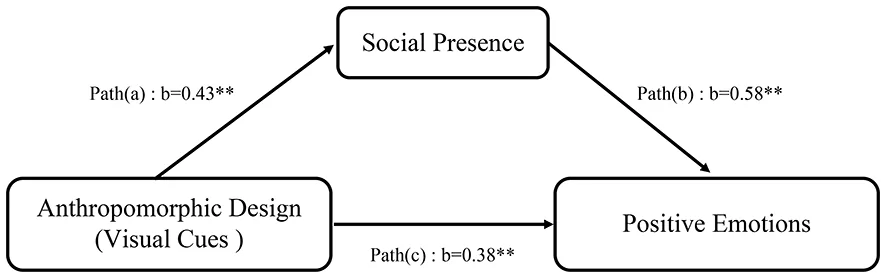
Mediating effect of social presence between anthropomorphic visual cues and positive emotions.

To further examine the mediating role of social presence in the relationship between anthropomorphic design (visual cues) and students' positive emotions, a bootstrap analysis was conducted following the recommended bootstrap procedure. Results showed that anthropomorphic design significantly predicted higher social presence (b = 0.43, *p* < 0.01), and social presence, in turn, significantly enhanced positive emotions (b = 0.58, *p* < 0.01). The direct effect of anthropomorphic design on positive emotions also remained significant (b = 0.38, *p* < 0.01). Furthermore, the indirect effect was significant, with the 95% confidence interval not including zero, indicating that social presence mediated the relationship between anthropomorphic design and positive emotions (see Figure 2). Thus, hypothesis H3a was supported.

#### 4.1.7 Discuss

The findings of Study 1a showed that incorporating anthropomorphic visual cues into virtual learning assistants, such as friendly facial expressions, and playful postures, significantly enhanced students' positive emotions and perceived social presence. More importantly, mediation analysis revealed that the effect of these visual cues on positive emotions operated through increased social presence. This suggests that human-like visual features not only alter the appearance of virtual learning assistants but also enhance their perceived social qualities, thereby increasing social presence and fostering more positive emotional experiences.

These findings contribute to the literature on anthropomorphic design., prior studies have primarily examined anthropomorphism in contexts such as brand image (Ma et al., 2023), product packaging (Ding et al., 2021), and service robots s (Dang and Liu, 2023) with a primary focus on consumer evaluations, attitudes, and behavioral responses. Study 1a broadens this literature to virtual learning environments by demonstrating that anthropomorphic design also shapes students' immediate emotional experiences during interactions with virtual learning assistants.

Furthermore, this findings extend Emotional Contagion Theory to virtual learning contexts by suggesting that emotional contagion processes may also operate in human–virtual agent interactions. This indicates that the affective influence of emotional cues is not necessarily confined to face-to-face interpersonal communication but can also emerge when users interact with virtual social agents (Herrando et al., 2022). The findings also support anthropomorphism theory by suggesting that human-like visual features encourage users to attribute human characteristics to virtual learning assistants, thereby eliciting social and emotional responses.

### 4.2 Study1b

Study 1b employed a one-factor between-subjects design (anthropomorphic verbal cues: anthropomorphic vs. non-anthropomorphic). We predicted that university students in the anthropomorphic verbal-cue condition would experience significantly higher levels of positive emotions than those in the non-anthropomorphic condition, and that social presence would mediate this relationship.

#### 4.2.1 Method and procedure

A total of 129 university students from Shenzhen, Guangdong, China participated in Study 1b (56% female; M = 21.83, SD = 3.81). Data were collection between February 20, and March 10, 2025. Participants received monetary compensation and were randomly distributed assigned to either the anthropomorphic and non-anthropomorphic design conditions. The experimental procedure was identical to that of Study 1a, except that anthropomorphic verbal cues were manipulated in the stimulus materials.

#### 4.2.2 Stimuli and manipulation

Study 1b used two versions of a mock knowledge-based learning platform interface presenting preset dialogues with a virtual learning assistant. In the anthropomorphic condition, the assistant communicated in a humorous and first-person manner. For example, when participants asked why they found a mathematics problem difficult to understand, the assistant responded, “I think these math problems might just be a little shy and do not want you to figure them out too quickly.” In the non-anthropomorphic condition, the assistant communicated in a neutral and impersonal manner. The two versions were identical in interface layout, visual appearance, color palette, font, and presentation format, differing only in verbal expressions. After the interaction, participants completed validated measures of perceived anthropomorphic design (verbal cues), social presence, and positive emotions using five-point Likert scales.

The experimental procedure followed that of Study 1a. After exposure to the stimuli, completed a four-item manipulation-check scale adapted from Chen et al. (2024) to assess perceived anthropomorphic verbal design. which has been widely applied in virtual agent contexts (Bartneck et al., 2009). The items were “The verbal style of the virtual learning assistant is natural,” “The verbal style of the virtual learning assistant is human-like,” “The verbal style of the virtual learning assistant is conscious,” and “The verbal style of the virtual learning assistant is elegant” (Cronbach's alpha = 0.871). The measures demonstrated satisfactory reliability, convergent validity, and discriminant validity. Detailed CR, AVE, and HTMT results are reported in the Supplementary material. Participants then completed the measures of social presence and positive emotions using the same scales described previously.

### 4.3 Result

#### 4.3.1 Manipulation check

An independent-samples *t*-test was conducted to examine whether perceived anthropomorphism differed between the anthropomorphic design (verbal cues) and non-anthropomorphic conditions. Results showed that participants in the anthropomorphic condition rated the assistant as significantly more human-like (M = 3.976, SD = 0.575) than those in the control condition (M = 2.814, SD = 1.048; t (127) = −6.967, *p* < 0.001, d = 1.271), confirming the validity of the anthropomorphic manipulation.

#### 4.3.2 Positive emotions

An independent-samples *t*-test showed that participants in the anthropomorphic design condition reported significantly higher positive emotion scores (M = 4.249, SD = 0.466) than those in the non-anthropomorphic condition (M = 2.531, SD = 0.959; t (127) = −12.392, *p* < 0.001, d = 2.278). thereby supporting Hypothesis H1b.

#### 4.3.3 Social presence

The analysis revealed that participants exposed to anthropomorphic verbal cues reported significantly greater social presence (M = 4.120, SD = 0.575) than those in the non-anthropomorphic condition (M = 3.105, SD = 1.116; t (127) = −6.612, *p* < 0.001, d = 1.144). These results confirm that anthropomorphic virtual learning assistant with verbal cues effectively enhanced students' perception of social presence, thereby supporting Hypothesis H2b.

#### 4.3.4 Mediation analysis

**Figure 3:**
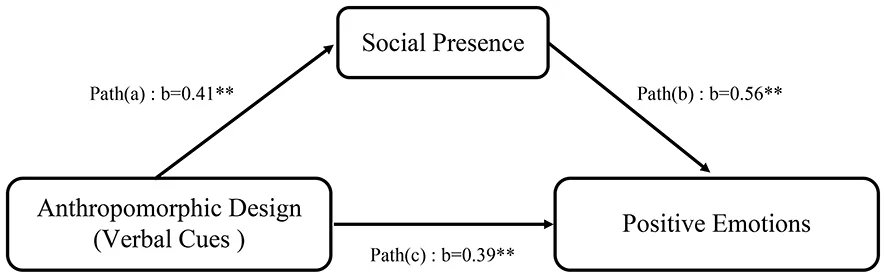
Mediating effect of social presence between anthropomorphic verbal cues and positive emotions.

The mediating role of social presence was examined using the bootstrap method. Results showed that anthropomorphic design significantly increased social presence (b = 0.41, *p* < 0.01), and social presence, in turn, significantly enhanced positive emotions (b = 0.56, *p* < 0.01). The total effect of anthropomorphic design on positive emotions was also significant (b = 0.39, *p* < 0.01). In addition, the indirect effect was significant, and the 95% confidence interval did not include zero, indicating that social presence mediated the relationship between anthropomorphic design and positive emotions (see Figure 3). Thus, Hypothesis H3b was supported.

### 4.4 Discuss

The results of Study 1b demonstrated that incorporating anthropomorphic verbal cues, particularly humorous, warm, and engaging expressions, into virtual learning assistants significantly enhanced students' perceptions of social presence and positive emotions. This finding is consistent with previous research, showing that human-like and positive communication by artificial intelligence can strengthen users' perceptions of social interaction and increase pleasure and trust (Yang et al., 2025a). Similarly, Letheren et al. (2017), suggested that anthropomorphic text in virtual environments can elicit positive affect through emotional contagion. Extending these findings, the present study demonstrates that emotionally expressive anthropomorphic verbal cues can foster positive emotional experiences in virtual learning environments.

Furthermore, the findings suggest that when virtual learning assistants use human-like verbal cues, students are more likely to perceive them as socially responsive interaction partners, thereby strengthening perceived social interaction and social presence. These findings provides further support for the Computers as Social Actors (CASA) framework (Pelau et al., 2021) which proposes that individuals tend to apply social norms and expectations derived from human–human interaction when responding to technologies that display social cues. Notably, however, human-like visual and verbal cues may also lead users to attribute empathy, intentions, or autonomy to AI systems, even when such responses are generated through predefined programs and computational mechanisms. Therefore, the emotional benefits of anthropomorphic design should be balanced against its potential ethical risks (Brännström et al., 2024).

### 4.5 Study 2a

Study 2a aimed to test Hypotheses H4a and H5a by examining whether loneliness moderates the effects of anthropomorphic design on social presence and positive emotions in virtual learning assistants. Specifically, we hypothesized that, compared with the non-anthropomorphic condition, the effects of anthropomorphic visual cues on social presence and positive emotions would vary according to students' levels of loneliness.

#### 4.5.1 Method and procedure

Study 2a employed a one-factor between-subjects experimental design in which anthropomorphic visual cues were manipulated (anthropomorphic vs. non-anthropomorphic), while loneliness was measured as a continuous individual-difference moderator. The study was conducted online via Wjx.com, a widely used data collection platform in China. Participants were recruited through multiple university-based channels, including student WeChat groups, campus mailing lists, and university online platforms. Eligibility criteria required participants to be currently enrolled university students, aged 18 years or older, and to provide informed consent prior to participation. A total of 311 participants completed the survey. After excluding 41 responses due to failed attention checks, excessively short completion times, or duplicate submissions, 270 valid responses were retained, representing 86.8% of the submitted responses (48.5% female; M = 23.8, SD = 3.3). Data were collected between March 20 and May 1, 2025.

Participants first completed a pre-exposure measure of loneliness using the three-item UCLA Loneliness Scale developed by Hughes et al. (2004), which has been validated in the context of anthropomorphism (Deng et al., 2023). The scale consists of three five-point Likert items referring to “a lack of companionship,” “ignored,” and “isolated from others” (Cronbach's α = 0.913). Participants were then randomly assigned to either the anthropomorphic or non-anthropomorphic visual-cue condition. Loneliness was treated as a continuous individual-difference moderator in the subsequent analyses.

#### 4.5.2 Stimuli and manipulation

Study 2a used two versions of a language-learning application interface as experimental stimuli. After completing a learning task, participants were presented with a task-completion screen displaying a success message and a crown-shaped character. In the anthropomorphic condition, the character incorporated human-like visual cues, including animated limb movements and a cheerful facial expression. In the non-anthropomorphic condition, participants viewed a conventional version without these human-like features. The two versions were identical in size, color, and image resolution, with only the anthropomorphic visual cues differing between conditions. After viewing the stimuli, participants completed a questionnaire measuring perceived anthropomorphic design (visual cues), social presence, and positive emotions, followed by demographic information.

### 4.6 Result

#### 4.6.1 Manipulation check

An independent-samples *t*-test was conducted to assess the effectiveness of the anthropomorphic design (visual cues) manipulation. Participants in the anthropomorphic condition perceived the virtual learning assistant as significantly more human-like (M = 4.124, SD = 0.686) than those in the non-anthropomorphic condition (M = 2.845, SD = 1.270), t (268) = −9.732, *p* < 0.001, d = 1.258. These results confirmed the effectiveness of the manipulation.

#### 4.6.2 Moderation analysis

A linear regression analysis was conducted to examine whether loneliness moderated the effect of anthropomorphic visual cues on positive emotions. Anthropomorphic visual cues, loneliness, and their interaction term were entered as predictors of positive emotions. The interaction between anthropomorphic visual cues and loneliness was significant [B = 0.79, SE = 0.151, t (266) = 5.24, *p* < 0.001, 95% CI (0.493, 1.087)], indicating that the effect of anthropomorphic visual cues on positive emotions varied as a function of loneliness.

To probe the interaction, conditional effects were examined at relatively low (−1 SD) and high (+1 SD) levels of loneliness. At a high level of loneliness (+1 SD), anthropomorphic visual cues had a significant positive effect on positive emotions [B = 0.83, SE = 0.109, t (266) = 7.59, p < 0.001, 95% CI (0.615, 1.045)], compared with that at a low level of loneliness (−1 SD) (B = 0.309, SE = 0.133, t (266) = 2.32, *p* = 0.021, 95% CI [0.047, 0.571]). These findings support Hypothesis H5a.

#### 4.6.3 Moderated mediation analysis

Following Hayes (2013), anthropomorphic visual cues (anthropomorphic = 1; non-anthropomorphic = 0) were entered as the independent variable, loneliness as a continuous moderator, social presence as the mediator, and positive emotions as the dependent variable. The interaction between anthropomorphic visual cues and loneliness significantly predicted social presence (B = 1.52, SE = 0.253, t (266) = 6.00, *p* < 0.001, 95% CI [1.021, 2.019]), indicating that loneliness moderated the effect of anthropomorphic visual cues on social presence.

Social presence, in turn, significantly predicted positive emotions (B = 0.37, SE = 0.06, t (267) = 6.17, *p* < 0.001, 95% CI [0.252, 0.488]). Bootstrap analyses based on 5,000 resamples were conducted to estimate the conditional indirect effects of anthropomorphic visual cues on positive emotions through social presence at different levels of loneliness. At a high level of loneliness (+1 SD), the indirect effect was significant [95% CI (0.521, 0.982)], whereas at a low level of loneliness (−1 SD), the indirect effect was not significant [95% CI (−0.063, 0.057)]. Moreover, the index of moderated mediation was significant, as its 95% confidence interval did not include zero [(0.306, 0.819)]. These findings indicate that the indirect effect of anthropomorphic visual cues on positive emotions through social presence became stronger as loneliness increased, supporting H4a.

### 4.7 Discuss

The findings of Study 2a showed that loneliness strengthened the effect of anthropomorphic visual cues on social presence and amplified their indirect effect on positive emotions through social presence. Students with higher levels of loneliness may be more sensitive to human-like facial features, expressions, and postures, making them more likely to perceive virtual learning assistants as socially present interaction partners. This finding differs from Yamaguchi et al. (2023), who reported that loneliness did not significantly influence users' attitudes toward or acceptance of anthropomorphic technologies. One possible explanation is that virtual learning assistants inherently involve stronger social interaction, allowing students with higher levels of loneliness to derive a greater sense of social connection from anthropomorphic visual cues.

Furthermore, this finding supports the applicability of Social Surrogacy Theory to virtual agent contexts. For students with higher levels of loneliness, human-like visual features may make virtual learning assistants appear less like purely functional tools and more like social interaction partners. Social presence plays a key role in this process by strengthening the perceived social nature of the interaction, thereby enhancing positive emotions. This interpretation is consistent with Zhang et al. (2023), who suggested that anthropomorphic virtual interactions can partially simulate interpersonal experiences and serve a social surrogate function.

### 4.8 Study 2b

Study 2b aimed to test Hypotheses H4b and H5b by examining whether loneliness moderates the effects of anthropomorphic design on social presence and positive emotions in virtual learning assistants. Specifically, we hypothesized that, compared with the non-anthropomorphic condition, the effects of anthropomorphic verbal cues on social presence and positive emotions would vary according to students' levels of loneliness.

#### 4.8.1 Method and procedure

Study 2b employed a one-factor between-subjects design in which anthropomorphic verbal cues were manipulated (anthropomorphic vs. non-anthropomorphic), with loneliness measured as a continuous moderator. Participants were recruited online through Wjx.com using university-based channels similar to those employed in Study 2a. A total of 230 university students completed the survey. Following the same data-screening procedure, 224 valid responses were retained (49.1% female; M = 23.0, SD = 3.62). Data were collected between May 20 and June 30, 2025.

Participants first completed the same loneliness measure used in Study 2a and were then randomly assigned to either the anthropomorphic or non-anthropomorphic verbal-cue condition. Loneliness was treated as a continuous moderator in the subsequent analyses.

#### 4.8.2 Stimuli and manipulation

Study 2b used two versions of an online classroom interface to manipulate anthropomorphic verbal cues. In the anthropomorphic condition, the virtual learning assistant (VLA) communicated with participants using emotionally expressive, first-person language and human-like verbal expressions (e.g., “Are you ready? I'm your personal learning assistant. Let's dive into a brand-new learning journey together! I'm sure you'll be even better than you were yesterday!”). In the non-anthropomorphic condition, the VLA delivered comparable information using neutral, objective, and impersonal language (e.g., “Please complete your learning task. Upon completion, the system will unlock the next stage of the course for you.”). The two versions were identical in interface layout and presentation format, with only the verbal cues differing between conditions. After viewing the stimuli, participants completed measures of perceived anthropomorphic design (verbal cues), social presence, and positive emotions, followed by demographic information.

### 4.9 Result

#### 4.9.1 Manipulation check

An independent-samples *t*-test was conducted to assess the effectiveness of the anthropomorphic design (verbal cues) manipulation. Participants in the anthropomorphic condition perceived the virtual learning assistant as significantly more human-like (M = 4.104, SD = 0.729) than those in the non-anthropomorphic condition (M = 2.729, SD = 1.258), t (222) = −9.073, *p* < 0.001, d = 1.329. These results confirmed the effectiveness of the manipulation.

#### 4.9.2 Moderation analysis

A linear regression analysis was conducted to examine whether loneliness moderated the effect of anthropomorphic verbal cues on positive emotions. Anthropomorphic verbal cues, loneliness, and their interaction term were entered as predictors of positive emotions. The interaction between anthropomorphic verbal cues and loneliness was significant (B = 0.93, SE = 0.174, t (220) = 5.33, *p* < 0.001, 95% CI [0.586, 1.274]), indicating that the effect of anthropomorphic verbal cues on positive emotions varied as a function of loneliness.

To probe the interaction, conditional effects were examined at relatively low (−1 SD) and high (+1 SD) levels of loneliness. At a high level of loneliness (+1 SD), anthropomorphic verbal cues had a significant positive effect on positive emotions (B = 1.759, SE = 0.217, t (220) = 8.11, *p* < 0.001, 95% CI [1.332, 2.186]), compared with the effect at a low level of loneliness (−1 SD) (B = 0.50, SE = 0.17, t (220) = 2.94, p =0.004, 95% CI [0.17, 0.83]). The positive effect of anthropomorphic verbal cues was stronger at higher levels of loneliness, supporting Hypothesis H5b.

#### 4.9.3 Moderated mediation analysis

Following Hayes (2013), anthropomorphic verbal cues (anthropomorphic = 1; non-anthropomorphic = 0) were entered as the independent variable, loneliness as a continuous moderator, social presence as the mediator, and positive emotions as the dependent variable. The interaction between anthropomorphic verbal cues and loneliness significantly predicted social presence (B = 1.64, SE = 0.28, t (220) = 5.86, *p* < 0.001, 95% CI [1.088, 2.192]), indicating that loneliness moderated the effect of anthropomorphic verbal cues on social presence.

Social presence, in turn, significantly predicted positive emotions (B = 0.37, SE = 0.07, t (221) = 5.29, *p* < 0.001, 95% CI [0.232, 0.508]). Bootstrap analyses based on 5,000 resamples were conducted to estimate the conditional indirect effects of anthropomorphic verbal cues on positive emotions through social presence at different levels of loneliness. At a high level of loneliness (+1 SD), the indirect effect was significant [95% CI (0.301, 0.548)], whereas at a low level of loneliness (−1 SD), the indirect effect was not significant [95% CI (−0.035, 0.118)]. Moreover, the index of moderated mediation was significant, with its 95% bootstrap confidence interval excluding zero [(0.380, 0.840)]. These findings indicate that the indirect effect of anthropomorphic verbal cues on positive emotions through social presence became stronger as loneliness increased, supporting Hypothesis H4b.

### 4.10 Discuss

The findings of Study 2b show that students' responses to anthropomorphic verbal cues in virtual learning assistants vary with loneliness. Students with higher levels of loneliness may be more attentive to social information conveyed through warmth, humor, and conversational expressions, making them more likely to perceive the assistant as a socially present interaction partner. This finding is consistent with previous research showing that linguistic cues can convey social meaning and shape perceptions of technological agents (Rao Hill and Troshani, 2024). Extending this work, the present study suggests that anthropomorphic verbal cues may be particularly effective in fostering social connection and positive emotional responses among students experiencing greater loneliness.

Furthermore, the present study extends Social Presence Theory by identifying loneliness as an important boundary condition in responses to anthropomorphic verbal communication. While Social Presence Theory primarily emphasizes how mediated cues create a sense of interacting with a social other, the present study further shows that the effectiveness of these cues depends on users' psychological characteristics. In virtual learning contexts, anthropomorphic verbal expressions can make otherwise instrumental interactions more closely resemble interpersonal communication. Social presence, in turn, serves as the psychological mechanism through which these social signals contribute to positive emotional experiences.

## 5 Research conclusions and implications

### 5.1 Conclusion

This study examined how anthropomorphic virtual learning assistants (VLAs) influence university students' positive emotions and whether these effects vary according to students' levels of loneliness. Across four experimental studies, anthropomorphic design, through both visual and verbal cues, elicited stronger positive emotions than non-anthropomorphic design.

Specifically, Study 1a showed that anthropomorphic visual cues enhanced students' positive emotions, with social presence serving as a significant mediator. Study 1b replicated this pattern for anthropomorphic verbal cues, demonstrating that their positive emotional effects were also mediated by increased social presence. Studies 2a and 2b further identified loneliness as an important individual-difference boundary condition. Loneliness strengthened the relationships between anthropomorphic visual and verbal cues and social presence, with students reporting greater loneliness showing stronger responses to these anthropomorphic cues. The indirect effects of anthropomorphic design on positive emotions through social presence also became stronger as loneliness increased.

Taken together, these findings demonstrate that anthropomorphic visual and verbal cues can enhance students' positive emotional experiences by increasing social presence, while the strength of these responses varies according to students' levels of loneliness. The study thus highlights social presence as an important psychological mechanism underlying the emotional effects of anthropomorphic VLAs and loneliness as a meaningful boundary condition shaping these effects. Rather than suggesting that anthropomorphic VLAs reduce loneliness itself, the findings indicate that students experiencing greater loneliness may be particularly responsive to the social cues embedded in anthropomorphic virtual learning assistants.

### 5.2 Theoretical implications

This study makes three theoretical contributions to research on anthropomorphic virtual learning assistants and students' emotional experiences. First, it moves beyond a direct-effect account of anthropomorphic design by identifying social presence as a key psychological mechanism. Human-like visual and verbal cues enhanced students' positive emotions partly by increasing the perceived social presence, warmth, and interpersonal responsiveness of the VLA. This finding integrates anthropomorphism, social response, and emotional contagion perspectives and clarifies how anthropomorphic design influences positive emotional experiences in virtual learning environments.

Second, the findings suggest that visual and verbal anthropomorphic cues may activate a common social-perceptual process despite their different forms. Human-like expressions and movements, as well as warm, humorous, and responsive language, increased social presence and positive emotions in the respective studies. The theoretical relevance of anthropomorphic cues may therefore lie in their shared capacity to signal social responsiveness and evoke interpersonal perceptions. Because visual and verbal cues were examined separately, the findings do not establish their relative effectiveness but provide converging evidence across both cue types.

Third, this study clarifies loneliness as an individual boundary condition rather than an outcome reduced by anthropomorphic VLAs. Students experiencing higher levels of loneliness showed stronger social presence and positive emotional responses to anthropomorphic cues, suggesting that unmet needs for social connection may increase sensitivity to socially responsive technologies. This finding refines Social Surrogacy Theory by explaining for whom anthropomorphic agents are more emotionally effective, without implying that they directly alleviate loneliness.

Together, these findings advance the theoretical understanding of anthropomorphic VLA design by clarifying the psychological mechanism through which it influences positive emotions, providing converging evidence across visual and verbal cues, and identifying loneliness as an important boundary condition.

### 5.3 Practical implications

This study provides practical implications for the responsible design and implementation of virtual learning assistants (VLAs). The appropriate use of anthropomorphic visual and verbal cues, such as human-like expressions, warm communication, and responsive language, may enhance students' social presence, emotional engagement, and immediate positive emotional experiences, thereby improving the interactivity and affective quality of virtual learning environments.

However, the positive emotional effects of anthropomorphic design may also raise potential ethical concerns. As Brännström et al. (2024) noted, users' perceptions may arise from how AI systems are designed and presented rather than from any genuine subjective emotions possessed by the systems themselves. Although such perceptions may enhance social presence and positive emotions, they may also blur the boundary between simulated emotional responses and genuine empathy. Danaher (2020)further argued that highly human-like expressions may lead users to overestimate AI systems' emotional understanding or autonomy, potentially making such designs deceptive. Similarly, Luger and Sellen (2016) found that mismatches between users' expectations and the actual capabilities of intelligent agents may undermine trust. Therefore, the positive emotional effects observed in this study should be understood as users' psychological responses to anthropomorphic cues rather than as evidence that VLAs possess genuine empathy, emotional understanding, or human-like autonomy.

Particular caution is required when anthropomorphic VLAs are used by students experiencing higher levels of loneliness. Although these students may respond more positively to cues of warmth, care, and social presence, they may also be more likely to overestimate the system's emotional capacities or become overly reliant on artificial interaction. Accordingly, VLAs should be positioned as supportive educational technologies rather than substitutes for interpersonal relationships, teacher and peer support, university counseling, or professional psychological services. Responsible implementation should therefore balance positive emotional experiences with transparency, appropriately calibrated anthropomorphism, user autonomy, and access to human support.

### 5.4 Limitations and future research

Despite its theoretical and practical contributions, this study has several limitations that suggest directions for future research.

First, the experimental stimuli mainly consisted of static icons, interface information, and brief verbal scripts. Thus, the findings primarily reflect students' immediate responses to anthropomorphic VLA prototypes rather than their experiences with fully functional assistants capable of sustained dialogue and adaptive feedback. Future research could use more dynamic stimuli in authentic learning tasks and examine outcomes such as learning engagement, performance, and continued use. In addition, this study focused on visual and verbal anthropomorphic cues, leaving features such as vocal tone, behavioral feedback, response style, and adaptive interaction unexplored. Future studies could compare different types and combinations of anthropomorphic cues.

Second, this study mainly relied on single-session experiments and self-report measures of immediate emotional responses, limiting its ability to capture long-term changes. Loneliness was also measured as a continuous individual-difference variable rather than experimentally manipulated. Future research could adopt longitudinal or repeated-measures designs and incorporate behavioral, physiological, and learning-performance indicators.

Third, the relatively limited sample size may restrict the generalizability of the findings to the broader population of Chinese university students. Future research should recruit participants from more diverse regional, cultural, and educational backgrounds.

Finally, this study focused on social presence as a mediator and loneliness as a moderator. Future research could examine other individual-difference factors, such as personality traits, cultural background, perceived social support, and anthropomorphic tendencies, to further clarify the mechanisms and boundary conditions underlying students' emotional responses to anthropomorphic VLAs.

## Data Availability

The original contributions presented in the study are included in the article/Supplementary material, further inquiries can be directed to the corresponding author.

## References

[B1] Adam M. Wessel M. Benlian A. (2021). AI-based chatbots in customer service and their effects on user compliance. Electron Mark. 31, 427–445. doi: 10.1007/s12525-020-00414-7

[B2] Affandi S. Ishaq M. I. Raza A. Talpur Q. Ahmad R. (2025). AI assistant is my new best friend! Role of emotional disclosure, performance expectations and intention to reuse. J. Retail. Consumer Serv. 82:104087. doi: 10.1016/j.jretconser.2024.104087

[B3] Aggarwal P. McGill A. L. (2007). Is That Car Smiling at Me? Schema congruity as a basis for evaluating anthropomorphized products. J. Consumer Res. 34, 468–479. doi: 10.1086/518544

[B4] Alabed A. Javornik A. Gregory-Smith D. (2022). AI anthropomorphism and its effect on users' self-congruence and self–AI integration: a theoretical framework and research agenda. Technol. Forecast. Soc. Change 182:121786. doi: 10.1016/j.techfore.2022.121786

[B5] An P. Zhang C. Gao H. Zhou Z. Xiao Y. Zhao J. (2025). AniBalloons: Animated chat balloons as affective augmentation for social messaging and chatbot interaction. Int. J. Hum. Comput. Stud. 194:103365. doi: 10.1016/j.ijhcs.2024.103365

[B6] Aristovnik A. KerŽič D. Ravšelj D. TomaŽevič N. Umek L. (2020). Impacts of the COVID-19 pandemic on life of higher education students: a global perspective. Sustainability 12:8438. doi: 10.3390/su12208438 PMC8634691 34869802

[B7] Barjaková M. Garnero A. d'Hombres B. (2023). Risk factors for loneliness: a literature review. Soc. Sci. Med. 334:116163. doi: 10.1016/j.socscimed.2023.116163 PMC10523154 37625251

[B8] Bartneck C. Kulić D. Croft E. Zoghbi S. (2009). Measurement instruments for the anthropomorphism, animacy, likeability, perceived intelligence, and perceived safety of robots. Int. J. Soc. Robot. 1, 71–81. doi: 10.1007/s12369-008-0001-3

[B9] Berridge C. Zhou Y. Robillard J. M. Kaye J. (2023). Companion robots to mitigate loneliness among older adults: Perceptions of benefit and possible deception. Front. Psychol. 14:1106633. doi: 10.3389/fpsyg.2023.1106633 36895732 PMC9988932

[B10] Biocca F. Harms C. Burgoon J. K. (2003). Toward a more robust theory and measure of social presence: review and suggested criteria. Presence Teleoperat. Virt. Environ. 12, 456–480. doi: 10.1162/105474603322761270

[B11] Brännström A. Wester J. Nieves J. C. (2024). A formal understanding of computational empathy in interactive agents. Cogn. Syst. Res. 85:101203. doi: 10.1016/j.cogsys.2023.101203

[B12] Chen J. Li M. Ham J. (2024). Different dimensions of anthropomorphic design cues: how visual appearance and conversational style influence users' information disclosure tendency towards chatbots. Int. J. Hum. Comput. Stud. 190:103320. doi: 10.1016/j.ijhcs.2024.103320

[B13] Chen L. Wang L. Qiu X. H. Yang X. X. Qiao Z. X. Yang Y. J. . (2013). Depression among Chinese University students: prevalence and socio-demographic correlates. PLoS ONE 8:e58379. doi: 10.1371/journal.pone.0058379 23516468 PMC3596366

[B14] Chen R. P. Wan E. W. Levy E. (2017). The effect of social exclusion on consumer preference for anthropomorphized brands. J. Consum. Psychol. 27, 23–34. doi: 10.1016/j.jcps.2016.05.004

[B15] Danaher J. (2020). Robot betrayal: a guide to the ethics of robotic deception. Ethics Inf. Technol. 22, 117–128. doi: 10.1007/s10676-019-09520-3

[B16] Dang J. Liu L. (2023). Do lonely people seek robot companionship? A comparative examination of the Loneliness–Robot anthropomorphism link in the United States and China. Comput. Hum. Behav. 141:107637. doi: 10.1016/j.chb.2022.107637

[B17] Deng F. Lin Y. Jiang X. (2023). Influence mechanism of consumers' characteristics on impulsive purchase in E-commerce livestream marketing. Comput. Human Behav. 148:107894. doi: 10.1016/j.chb.2023.107894

[B18] Diehl K. Jansen C. Ishchanova K. Hilger-Kolb J. (2018). Loneliness at Universities: determinants of emotional and social loneliness among students. Int. J. Environ. Res. Public Health 15:1865. doi: 10.3390/ijerph15091865 30158447 PMC6163695

[B19] Ding Z. Sun J. Wang Y. Jiang X. Liu R. Sun W. . (2021). Research on the influence of anthropomorphic design on the consumers' express packaging recycling willingness:the moderating effect of psychological ownership. Res. Conserv. Recycl. 168:105269. doi: 10.1016/j.resconrec.2020.105269

[B20] Ellard O. B. Dennison C. Tuomainen H. (2023). Review: interventions addressing loneliness amongst university students: a systematic review. Child Adolesc. Ment. Health 28, 512–523. doi: 10.1111/camh.12614 36496554

[B21] Epley N. Waytz A. Cacioppo J. T. (2007). On seeing human: a three-factor theory of anthropomorphism. Psychol. Rev. 114, 864–886. doi: 10.1037/0033-295X.114.4.864 17907867

[B22] Feng W. (2016). When lonely people encounter anthropomorphic products. Soc. Behav. Personal. Int. J. 44, 1649–1660. doi: 10.2224/sbp.2016.44.10.1649

[B23] Fredrickson B. L. (2000). Cultivating positive emotions to optimize health and wellbeing. Prevent. Treat. 3:31 doi: 10.1037/1522-3736.3.1.31a

[B24] Gao W. Liu Z. Li J. (2017). How does social presence influence SNS addiction? A belongingness theory perspective. Comput. Hum. Behav. 77, 347–355. doi: 10.1016/j.chb.2017.09.002

[B25] Gubareva R. Lopes R. (2020). “Virtual assistants for learning: a systematic literature review,” in Proceedings of the 12th International Conference on Computer Supported Education. 12th International Conference on Computer Supported Education. Prague: Czech Republic. doi: 10.5220/0009417600970103

[B26] Han E. Yin D. Zhang H. (2023). Bots with feelings: should AI agents express positive emotion in customer service? Inform. Syst. Res. 34, 1296–1311. doi: 10.1287/isre.2022.1179

[B27] Haugeland I. K. F. Følstad A. Taylor C. Bjørkli C. A. (2022). Understanding the user experience of customer service chatbots: an experimental study of chatbot interaction design. Int. J. Hum. Comput. Stud. 161:102788. doi: 10.1016/j.ijhcs.2022.102788

[B28] Hayes A. F. (2013). Introduction to Mediation, Moderation, and Conditional Process Analysis: A Regression-Based Approach. New York, NY: The Guilford Press.

[B29] Heerink M. Kröse B. Evers V. Wielinga B. (2010). Assessing acceptance of assistive social agent technology by older adults: the almere model. Int. J. Soc. Robot. 2, 361–375. doi: 10.1007/s12369-010-0068-5

[B30] Herbener A. B. Damholdt M. F. (2025). Are lonely youngsters turning to chatbots for companionship? The relationship between chatbot usage and social connectedness in Danish high-school students. Int. J. Hum. Comput. Stud. 196:103409. doi: 10.1016/j.ijhcs.2024.103409

[B31] Herrando C. Constantinides E. (2021). Emotional contagion: a brief overview and future directions. Front. Psychol. 12:712606. doi: 10.3389/fpsyg.2021.712606 34335425 PMC8322226

[B32] Herrando C. Jiménez-Martínez J. Martín-De Hoyos M. J. Constantinides E. (2022). Emotional contagion triggered by online consumer reviews: Evidence from a neuroscience study. J. Retail. Consum. Serv. 67:102973. doi: 10.1016/j.jretconser.2022.102973

[B33] Hickin N. Käll A. Shafran R. Sutcliffe S. Manzotti G. Langan D. (2021). The effectiveness of psychological interventions for loneliness: a systematic review and meta-analysis. Clin. Psychol. Rev. 88:102066. doi: 10.1016/j.cpr.2021.102066 34339939

[B34] Hsieh S. H. Lee C. T. Tseng T. H. (2021). Branded app atmospherics: examining the effect of pleasure–arousal–dominance in brand relationship building. J. Retail. Consum. Serv. 60:102482. doi: 10.1016/j.jretconser.2021.102482

[B35] Hughes M. E. Waite L. J. Hawkley L. C. Cacioppo J. T. (2004). A short scale for measuring loneliness in large surveys: results from two population-based studies. Res. Aging 26, 655–672. doi: 10.1177/0164027504268574 18504506 PMC2394670

[B36] Indrayanti I. Salsabila A. K. Amrita V. Alhaddad M. M. Saskia A. B. Ramadhani D. P. (2025). PsyBot: a randomized controlled trial of WhatsApp-based psychological first aid to reduce loneliness among 18–22-year-old students in Yogyakarta, Indonesia. SSM Ment. Health 8:100504. doi: 10.1016/j.ssmmh.2025.100504

[B37] Janson A. (2023). How to leverage anthropomorphism for chatbot service interfaces: the interplay of communication style and personification. Comput. Human Behav. 149:107954. doi: 10.1016/j.chb.2023.107954

[B38] Jeong H. J. Kim J. Chung D. S. (2022). Being present as ‘real' humans on social media: how do personified brand visuals lead to consumer engagement? Visual Commun. Q. 29, 236–249. doi: 10.1080/15551393.2022.2129656

[B39] Jiang Y. Yang X. Zheng T. (2023). Make chatbots more adaptive: dual pathways linking human-like cues and tailored response to trust in interactions with chatbots. Comput. Human Behav. 138:107485. doi: 10.1016/j.chb.2022.107485

[B40] Khan F. M. Anas M. Uddin S. M. F. (2023). Anthropomorphism and consumer behaviour: a SPAR-4-SLR protocol compliant hybrid review. Int. J. Consum. Stud. 49:12985. doi: 10.1111/ijcs.12985

[B41] Kim J. Merrill K. Xu K. Sellnow D. D. (2021). I like my relational machine teacher: an ai instructor's communication styles and social presence in online education. Int. J. Hum. Comput. Interact. 37, 1760–1770. doi: 10.1080/10447318.2021.1908671

[B42] Kim T. Sung Y. Moon J. H. (2020). Effects of brand anthropomorphism on consumer-brand relationships on social networking site fan pages: the mediating role of social presence. Telemat. Inform. 51:101406. doi: 10.1016/j.tele.2020.101406

[B43] Konya-Baumbach E. Biller M. Von Janda S. (2023). Someone out there? A study on the social presence of anthropomorphized chatbots. Comput. Hum. Behav. 139:107513. doi: 10.1016/j.chb.2022.107513

[B44] Le T. P. Cowan T. Schwartz E. K. Elvevåg B. Holmlund T. B. Foltz P. W. . (2019). The importance of loneliness in psychotic-like symptoms: data from three studies. Psychiatry Res. 282:112625. doi: 10.1016/j.psychres.2019.112625 31662188

[B45] Letheren K. Martin B. A. S. Jin H. S. (2017). Effects of personification and anthropomorphic tendency on destination attitude and travel intentions. Tour. Manag. 62, 65–75. doi: 10.1016/j.tourman.2017.03.020

[B46] Li L. Chen X. Zhu P. (2024a). How do e-commerce anchors' characteristics influence consumers' impulse buying? An emotional contagion perspective. J. Retail. Consum. Serv. 76:103587. doi: 10.1016/j.jretconser.2023.103587

[B47] Li M. Wang R. (2023). Chatbots in e-commerce: the effect of chatbot language style on customers' continuance usage intention and attitude toward brand. J. Retail. Consum. Serv. 71:103209. doi: 10.1016/j.jretconser.2022.103209

[B48] Li N. Xuan C. Chen R. (2024b). Different roles of two kinds of digital coexistence: the impact of social presence on consumers' purchase intention in the live streaming shopping context. J. Retail. Consum. Serv. 80:103890. doi: 10.1016/j.jretconser.2024.103890

[B49] Lim X.-J. Cheah J.-H. Ng S. I. Basha N. K. Soutar G. (2021). The effects of anthropomorphism presence and the marketing mix have on retail app continuance use intention. Technol. Forecast. Soc. Change 168:120763. doi: 10.1016/j.techfore.2021.120763

[B50] Lin J.-S. (2025). Examining the interplay of chatbot interactivity and anthropomorphic design for enhancing brand relationships in service businesses. Int. J. Advert. 44, 829–860. doi: 10.1080/02650487.2024.2410559

[B51] Liu W. Jiang M. Li W. Mou J. (2024). How does the anthropomorphism of AI chatbots facilitate users' reuse intention in online health consultation services? The moderating role of disease severity. Technol. Forecast. Soc. Change 203:123407. doi: 10.1016/j.techfore.2024.123407

[B52] Luger E. Sellen A. (2016). “'Like having a really bad PA': the gulf between user expectation and experience of conversational agents,” in Proceedings of the 2016 CHI Conference on Human Factors in Computing Systems, 5286–5297. doi: 10.1145/2858036.2858288

[B53] Ma J. Tu H. Zhou X. Niu W. (2023). Can brand anthropomorphism trigger emotional brand attachment? Serv. Ind. J. 43, 555–578. doi: 10.1080/02642069.2021.2012163

[B54] Mayor Poupis L. Rubin D. Lteif L. (2021). Turn up the volume if you're feeling lonely: the effect of mobile application sound on consumer outcomes. J. Bus. Res. 126, 263–278. doi: 10.1016/j.jbusres.2020.12.062

[B55] Miao F. Kozlenkova I. V. Wang H. Xie T. Palmatier R. W. (2022). An emerging theory of avatar marketing. J. Mark. 86, 67–90. doi: 10.1177/0022242921996646

[B56] Mishra R. Mehta R. (2023). The effects of food anthropomorphism on consumer behavior: a systematic literature review with integrative framework and future research directions. Appetite 190:107035. doi: 10.1016/j.appet.2023.107035 37704008

[B57] Mizani H. Cahyadi A. Hendryadi H. Salamah S. Retno Sari S. (2022). Loneliness, student engagement, and academic achievement during emergency remote teaching during COVID-19: the role of the God locus of control. Humanit. Soc. Sci. Commun. 9:305. doi: 10.1057/s41599-022-01328-9 36105275 PMC9461404

[B58] Munnukka J. Talvitie-Lamberg K. Maity D. (2022). Anthropomorphism and social presence in Human–Virtual service assistant interactions: the role of dialog length and attitudes. Comput. Hum. Behav. 135:107343. doi: 10.1016/j.chb.2022.107343

[B59] Nass C. Moon Y. (2000). Machines and mindlessness: social responses to computers. J. Soc. Issues 56, 81–103. doi: 10.1111/0022-4537.00153

[B60] Park C. Majeed A. Gill H. Tamura J. Ho R. C. Mansur R. B. . (2020). The effect of loneliness on distinct health outcomes: a comprehensive review and meta-analysis. Psychiatr. Res. 294:113514. doi: 10.1016/j.psychres.2020.113514 33130511

[B61] Park G. Yim M. C. Chung J. Lee S. (2023). Effect of AI chatbot empathy and identity disclosure on willingness to donate: the mediation of humanness and social presence. Behav. Inform. Technol. 42, 1998–2010. doi: 10.1080/0144929X.2022.2105746

[B62] Pelau C. Dabija D.-C. Ene I. (2021). What makes an AI device human-like? The role of interaction quality, empathy and perceived psychological anthropomorphic characteristics in the acceptance of artificial intelligence in the service industry. Comput. Hum. Behav. 122:106855. doi: 10.1016/j.chb.2021.106855

[B63] Raisa A. Chen X. Bryan E. G. Bylund C. L. Alpert J. M. Lok B. . (2025). Virtual health Assistants in preventive cancer care communication: systematic review. JMIR Cancer 11:e73616–e73616. doi: 10.2196/73616 40953370 PMC12435786

[B64] Rao Hill S. Troshani I. (2024). Chatbot anthropomorphism, social presence, uncanniness and brand attitude effects. J. Comput. Inform. Syst. 1–17. doi: 10.1080/08874417.2024.2423187

[B65] Tian Y. Wang J. Zakaria S. F. Qiu Y. (2025a). “Lonely but not alone: how anthropomorphic virtual learning assistants enhance college students' positive emotions,” in 2025 14th International Conference on Educational and Information Technology (ICEIT), 423–428. doi: 10.1109/ICEIT64364.2025.10975869

[B66] Tian Y. Zakaria S. F. Du Y. Qiu Y. Tian Z. (2025b). Can anthropomorphic design in beverage packaging enhance impulse buying intention? Amazing visual and verbal cues*! PLOS One* 20:e0326186. doi: 10.1371/journal.pone.0326186 40522940 PMC12169521

[B67] Todericiu I. A. (2025). Virtual assistants: a review of the next frontier in AI Interaction. Acta Univ. Sapientiae, Informatica 17:7. doi: 10.1007/s44427-025-00002-7

[B68] Van Der Stap N. Van Den Bogaart T. Rahimi E. Versendaal J. (2024). Fostering online interaction in blended learning through social presence and convergence: a systematic literature review. J. Comput. Assist. Learn. 40, 1712–1726. doi: 10.1111/jcal.12981

[B69] Williamson S. Szocs C. (2021). Smiling faces on food packages can increase adults' purchase likelihood for children. Appetite 165:105301. doi: 10.1016/j.appet.2021.105301 33984403

[B70] Wu R. Liu J. Chen S. Tong X. (2023). The effect of E-commerce virtual live streamer socialness on consumers' experiential value: an empirical study based on Chinese E-commerce live streaming studios. J. Res. Interact. Market. 17, 714–733. doi: 10.1108/JRIM-09-2022-0265

[B71] Xie Y. Zhou P. Liang C. Zhao S. Lu W. (2025). Affiliative or self-defeating? Exploring the effect of humor types on customer forgiveness in the context of AI agents' service failure. J. Bus. Res. 194:115381. doi: 10.1016/j.jbusres.2025.115381

[B72] Xu K. Chen X. Huang L. (2022). Deep mind in social responses to technologies: a new approach to explaining the Computers are Social Actors phenomena. Comput. Human Behav. 134:107321. doi: 10.1016/j.chb.2022.107321

[B73] Yamaguchi M. Okanda M. Moriguchi Y. Itakura S. (2023). Young adults with imaginary companions: the role of anthropomorphism, loneliness, and perceived stress. Pers. Individ. Dif. 207:112159. doi: 10.1016/j.paid.2023.112159

[B74] Yan J. Xia S. Jiang A. Lin Z. (2024). The effect of different types of virtual influencers on consumers' emotional attachment. J. Bus. Res. 177:114646. doi: 10.1016/j.jbusres.2024.114646

[B75] Yang L. W. Aggarwal P. McGill A. L. (2020). The 3 C's of anthropomorphism: connection, comprehension, and competition. Consum. Psychol. Rev. 3, 3–19. doi: 10.1002/arcp.1054

[B76] Yang W. Xie Y. Wang Y. (2025a). Does the level of anthropomorphism induce cognitive crafting in human-robot interaction? The role of positive emotions and interaction frequency. J. Hospit. Market. Manag. 34, 549–573. doi: 10.1080/19368623.2025.2458602

[B77] Yang Y. Wang C. Xiang X. An R. (2025b). AI applications to reduce loneliness among older adults: a systematic review of effectiveness and technologies. Healthcare 13:446. doi: 10.3390/healthcare13050446 40077009 PMC11898439

[B78] Yu S. Zhao L. (2024). Emojifying chatbot interactions: an exploration of emoji utilization in human-chatbot communications. Telemat. Inform. 86:102071. doi: 10.1016/j.tele.2023.102071

[B79] Zhang W. Slade E. L. Pantano E. (2024). Humanlike service robots: a systematic literature review and research agenda. Psychol. Market. 41, 3157–3181. doi: 10.1002/mar.22099

[B80] Zhang Y. Li Y. Xia M. Han M. Yan L. Lian S. (2023). The relationship between loneliness and mobile phone addiction among Chinese college students: the mediating role of anthropomorphism and moderating role of family support. PLoS ONE 18:e0285189. doi: 10.1371/journal.pone.0285189 37115749 PMC10146513

[B81] Zhu W. Yan R. Ding Z. (2020). Analysing impulse purchasing in cross-border electronic commerce. Ind. Manag. Data Syst. 120, 1959–1974. doi: 10.1108/IMDS-01-2020-0046

