# Supplementary material for: Positive emotional responses to anthropomorphic virtual learning assistants among university students with different levels of loneliness
Source: Front Psychol. 2026 Sep 7;17:1758999. doi: 10.3389/fpsyg.2026.1758999 (PMC13591416; doi:10.3389/fpsyg.2026.1758999)
Supplement: Supplementary file 1 [file Data_Sheet_1.docx]

Supplementary Material

**Alleviating Loneliness among University Students through Anthropomorphic Virtual Learning Assistants: Insights from Positive Psychology**

Yuyang Tian and Ye Qiu*

*** Correspondence:** Corresponding Author: [qiuye53125050@163.com](mailto:qiuye53125050@163.com)

# 1 Supplementary Figures

**Supplementary Figure 1.** stimuli

Table S1. Comparison of Experimental Stimuli Across the Four Studies

| Study | Experimental Context | Manipulated Variable | Anthropomorphic Condition | Non-anthropomorphic Condition | Elements Held Constant | Presentation Duration |
| --- | --- | --- | --- | --- | --- | --- |
| Study 1a | Language translation application icon | Visual cues | Included human-like facial features (eyes, eyebrows, a smiling mouth), a thumbs-up gesture, and a headset with a microphone | Retained the same basic icon design but without human-like facial features | Icon size, color, background, position, and resolution | Self-paced reading |
| Study 1b | Knowledge-learning platform | Verbal cues | Used first-person expressions, self-introduction, direct forms of address, and warm and encouraging language | Used neutral, objective, and non-personalized system language, resulting in a neutral, non-anthropomorphic appearance | Interface layout, font, text position, task content, and resolution | Self-paced reading |
| Study 2a | Language-learning task completion interface | Visual cues | Included humorous facial expressions, dancing movements | Used the same interface but with a neutral expression | Success message, crown image, interface color, layout, and size | Self-paced reading |
| Study 2b | Online classroom interface | Verbal cues | Used first-person expressions, and emotionally expressive and encouraging language | Used neutral, objective, and standardized task instructions | Font, interface background, text position, task information, and resolution | Self-paced reading |

Table S2. Reliability and Convergent Validity

| Study | Construct | Items | Cronbach’s α | McDonald’s ω | CR | AVE |
| --- | --- | --- | --- | --- | --- | --- |
| Study 1a | Anthropomorphic Design（AD） | 4 | 0.871 | 0.890 | 0.900 | 0.691 |
|  | Social Presence (SP) | 5 | 0.841 | 0.857 | 0.902 | 0.648 |
|  | Positive Emotions (PE) | 6 | 0.912 | 0.885 | 0.905 | 0.705 |
| Study 1b | Anthropomorphic Design（AD） | 4 | 0.891 | 0.889 | 0.891 | 0.671 |
|  | Social Presence (SP) | 5 | 0.868 | 0.892 | 0.832 | 0.660 |
|  | Positive Emotions (PE) | 6 | 0.891 | 0.884 | 0.902 | 0.608 |
| Study 2a | Anthropomorphic Design（AD） | 4 | 0.819 | 0.792 | 0.881 | 0.612 |
|  | Loneliness (LN) | 3 | 0.913 | 0.835 | 0.912 | 0.711 |
|  | Social Presence (SP) | 5 | 0.901 | 0.871 | 0.877 | 0.674 |
|  | Positive Emotions (PE) | 6 | 0.826 | 0.845 | 0.850 | 0.686 |
| Study 2b | Anthropomorphic Design（AD） | 4 | 0.850 | 0.821 | 0.863 | 0.601 |
|  | Loneliness (LN) | 3 | 0.892 | 0.847 | 0.897 | 0.614 |
|  | Social Presence (SP) | 5 | 0.884 | 0.801 | 0.838 | 0.651 |
|  | Positive Emotions (PE) | 6 | 0.898 | 0.803 | 0.941 | 0.713 |

Table S3. Study 1a Heterotrait-Monotrait Ratio (HTMT)

| Study | Construct | AD | SP | PE |
| --- | --- | --- | --- | --- |
| Study 1a | Anthropomorphic Design（AD） |  |  |  |
|  | Social Presence (SP) | 0.501 |  |  |
|  | Positive Emotions (PE) | 0.496 | 0.466 |  |

Table S4. Study 1b Heterotrait-Monotrait Ratio (HTMT)

| Study | Construct | AD | SP | PE |
| --- | --- | --- | --- | --- |
| Study 1b | Anthropomorphic Design（AD） |  |  |  |
|  | Social Presence (SP) | 0.323 |  |  |
|  | Positive Emotions (PE) | 0.417 | 0.493 |  |

Table S5. Study 2a Heterotrait-Monotrait Ratio (HTMT)

| Study | Construct | AD | LN | SP | PE |
| --- | --- | --- | --- | --- | --- |
| Study 2a | Anthropomorphic Design（AD） |  |  |  |  |
|  | Loneliness (LN) | 0.302 |  |  |  |
|  | Social Presence (SP) | 0.143 | 0.312 |  |  |
|  | Positive Emotions (PE) | 0.434 | 0.219 | 0.378 |  |

Table S6. Study 2b Heterotrait-Monotrait Ratio (HTMT)

| Study | Construct | AD | LN | SP | PE |
| --- | --- | --- | --- | --- | --- |
| Study 2b | Anthropomorphic Design（AD） |  |  |  |  |
|  | Loneliness (LN) | 0.152 |  |  |  |
|  | Social Presence (SP) | 0.080 | 0.112 |  |  |
|  | Positive Emotions (PE) | 0.399 | 0.078 | 0.390 |  |
